# Mental health related stigma, service provision and utilization in Northern India: situational analysis

**DOI:** 10.1186/s13033-023-00577-8

**Published:** 2023-04-27

**Authors:** Amanpreet Kaur, Sudha Kallakuri, Ankita Mukherjee, Syed Shabab Wahid, Brandon A. Kohrt, Graham Thornicroft, Pallab K. Maulik

**Affiliations:** 1 Jindal School of Psychology & Counselling, O.P. Jindal Global University, Sonipat, India; 2 The George Institute for Global Health, Delhi, India; 3 Division of Global Mental Health, Department of Psychiatry and Behavioral Sciences, The George Washington University, Washington, DC USA; 4 Department of Global Health, Georgetown University, Washington, DC USA; 5 Centre for Global Mental Health and Centre for Implementation Science Health Service and Population Research Department, Institute of Psychiatry, Psychology & Neuroscience, King’s College London, De Crespigny Park, London, SE5 8AF UK; 6 University of New South Wales, Sydney, Ausralia Australia; 7 Prasanna School of Public Health, Manipal University, Manipal, India

**Keywords:** Stigma, Discrimination, Situational analysis, Mental health, Northern India

## Abstract

Stigma, discrimination, poor help seeking, dearth of mental health professionals, inadequate services and facilities all adversely impact the mental health treatment gap. Service utilization by the community is influenced by cultural beliefs and literacy levels. We conducted a situational analysis in light of the little information available on mental health related stigma, service provision and utilization in Haryana, a state in Northern India. This involved: (a) qualitative key informant interviews; (b) health facility records review; and (c) policy document review to understand the local context of Faridabad district in Northern India. Ethical approvals for the study were taken before the study commenced. Phone call in-depth interviews were carried out with a purposive sample of 13 participants (Mean = 38.07 years) during the COVID-19 pandemic, which included 4 community health workers, 4 people with mental illness, 5 service providers (primary health care doctors and mental health specialists). Data for health facility review was collected from local primary health and specialist facilities while key policy documents were critically analysed for service provision and stigma alleviation activities. Thematic analysis was used to analyse patterns within the interview data. We found poor awareness and knowledge about mental illnesses, belief in faith and traditional healers, scarcity of resources (medicines, trained professionals and mental health inpatient and outpatient clinics), poor access to appropriate mental health facilities, and high costs for seeking mental health care. There is a critical gap between mental health related provisions in policy documents and its implementation at primary and district level.

## Introduction

The treatment gap for people with mental illnesses is vast in low-and middle-income countries (LMIC) like India, and is estimated to be around 95% and only 1 in 20 receiving any treatment [1]. Research suggests multiple reasons for this treatment gap, including stigma, discrimination, poor awareness about mental illnesses, poor help seeking, lack of trained professionals and below-par availability of services across the country [2–4]. Stigma and discrimination against people with mental illness (PWMI) is ubiquitous and creates hurdles for mental health service utilization and help-seeking [5] which further contributes to the treatment gap for mental illnesses [6].

Despite being a public health concern, mental health has not received much priority in a country like India. India was one of the early LMIC to start a National Mental Health Programme (NMHP) way back in 1982, which aimed to: (a) ensure availability and accessibility of minimum mental health care, (b) encourage mental health knowledge and skills, and (c) promote community participation in mental health service development and stimulate self-help [7]. Many districts do not have minimum number of trained mental health professionals in primary and secondary care settings and irregular availability of psychotropic medicines [8]. To date, the NMHP has not been successful partly because of issues related to funding, lack of trained human resources and poor community participation [7, 9].

The availability of services is impacted by policies and programs. Poor utilization of services has roots in stigma, feared discrimination and embarrassment associated with mental illnesses in addition to lack of professionals trained in mental health and poorly resourced health care facilities which often do not provide time, space or privacy to the patients. Explanatory models of mental illnesses in India are shaped by Indian traditional medicine, supernatural beliefs, sins or bad deeds, faith/religious healers [1, 10]. Stigma is also maintained by service providers and their training, attitudes and knowledge play an important role in quality of mental health care delivery [5]. There is a critical gap between community understanding, professional help-seeking and implementation of mental health programs [4].

Accordingly, situational analysis is a critical first step in directing robust implementation research that can help promote improved availability of, access to, and reach of mental health services for people living with mental illness in LMICs [11]. In light of the limited information available on mental health related stigma, service provision and utilization in Haryana, a state in Northern India, the aim of this paper is to study the mental health related stigma, current gaps in mental health services and service utilization before planning for the implementation of an anti-stigma intervention.

## Methods

### Current study

The present study was carried out in Northern India as part of formative study which was nested within the larger INDIGO (International Study of Discrimination and Stigma Outcomes) Partnership Research Programme. The goals of the INDIGO study are to identify stigmatising language, behaviours, and institutional practices and their underlying mechanisms of action in stigmatisation processes across different cultural contexts in 5 countries - Ethiopia, China, India, Nepal, and Tunisia.

We conducted: (a) qualitative key informant interviews (KIIs), (b) health facility records review, and (c) policy document review, to understand the local context of Faridabad district in Haryana (a state in Northern India), the study implementation field site. Ethical approval for the study was taken from The Institute Ethics Committee and Indian Council of Medical Research.

### Context

Faridabad is the largest and most populous district in state of Haryana in Northern India. It is also situated in the National Capital Region bordering New Delhi. This district has a combination of rural, urban and semi-urban areas with a majority of the population residing in urban areas (https://faridabad.nic.in/demography/). There are 16 Primary Health Care Centres (primary care), one Government District Hospital (secondary care) and one sub-district level community outreach hospital (secondary care) in Faridabad. Mental health professionals are available only at the secondary care setting. The present analysis reports both primary and secondary data on the stigma related to mental health, existing policies, facilities and services for mental health, and other relevant local contextual factors.

### Qualitative key informant interviews

A total of 21 participants (> 18 years age, able to speak in English or Hindi languages and having access to mobile phones) were initially contacted through an ongoing mental health programme, Systematic Medical Appraisal, Referral and Treatment (SMART) Mental Health Project [12] in rural Haryana for qualitative KIIs. A total of 13 participants were recruited purposively, who had access to smartphones, and gave informed consent for audio-recordings of phone call in-depth interviews. Interviews were carried out till meaningful theoretical saturation was achieved [13]. The participants included – 4 community health workers (CHW) – ASHAs (Accredited Social Health Activists), and ASHA program coordinators (This cohort does not provide any medical or therapeutic services); 4 Service Users (SU) - people diagnosed with mental illness; and 5 Service Providers (SP) – 2 Primary Health Care (PHC) doctors with basic medical training and 3 mental health specialists (MHS) such as counsellors and psychiatrists from a secondary care hospital. The CHWs were actively involved with the field staff working in the SMART Mental Health Project in screening and referral of PWMI from the community. These CHWs and field staff played a key role in identifying the SUs for the present study. PHC doctors and MHS were in contact with the research team for this study.

These KIIs were carried out between 24th September and 28th October 2020. Due to the COVID-19 pandemic, face-to-face interaction restrictions and a nationwide lockdown in 2020, the initial plan to conduct in-person in-depth interviews was modified to phone call based in-depth interviews. The ASHAs from urban PHC settings could not be contacted for the same reason. The guidelines, instructions and interview guides were prepared both in English and Hindi languages by first and second author. Online consenting was done using WhatsApp, and records of each consent were maintained as per ethics guidelines. All interviews were audio recorded after getting consent from the participants. Notes were taken by two trained researchers in qualitative methodology (first and third author) for each participant and collated for analysis later.

The semi-structured interview guides were developed based on Kleinman’s explanatory model framework on mental illness [14]. The qualitative research was intended to inform anti-stigma interventions based on a cultural mechanism of “What matters most” [15, 16] and social contact theory [17, 18]. A prior version of the interview guide was used in 7 countries, including in a different site in India: Bengaluru [19]. The guides were modified contextually and adapted for local use after informal discussions with two service users and three service providers by first and second author. Field notes were also recorded while visiting both primary and secondary health care facilities. The discussions on the interview guides focused on clarity and adequacy of items, language used, types of probes being used, and relevance of questions. These five respondents were not part of the final set of respondents included during KIIs.

The domains included in the semi-structured interview guide were - cultural models of mental illness (knowledge and mental health literacy), engaging with PWMI, “what matters most” to healthcare workers and other stakeholder, detection of mental illness in primary care settings (attitudes towards help seeking), responding to experienced and anticipated discrimination (different experiences and responses related to mental illnesses), social contact with persons with mental illness (methods to reduce mental health related stigma), and health systems (for service providers and community health workers, facilities and resources available). Additional domains explored in the service users’ KIIs were their experience of mental health problems, help-seeking behaviours, experiences with service providers, experiences of stigma and discrimination, symptoms that were most stigmatized, and social contact (mental health related knowledge, attitudes, experiences and suggestions).

Apart from the interview guide, socio-demographic data was collected, including age, gender, level of education, marital status, work status, religious group, diagnosis, number of years in treatment, years of experience, level of training, and level of facility (primary, secondary or tertiary health care setting) in health system.

### Health facility record review

For the health facility record review, two Urban Primary Health Centres (UPHCs) were purposively selected. These were representative of typical primary care settings in Faridabad in functioning, services and resources. One district hospital included a specialist mental health setting. This secondary hospital catered to the needs of all referred cases from the Faridabad catchment area (both rural and urban), including the two UPHCs.

The record reviews for the afore-mentioned health facilities were conducted in April 2021. The records were cross-checked and discussed with the medical officers (SPs) or data operators at each site. The facility review included summary figures on - availability of assured mental health services, information and education materials (IEC), trained human resources, number of people treated for mental health conditions, management of mental illnesses, and physical infrastructure.

### Policy document review

The policy review consisted of scrutinizing publicly available key documents providing information on India specific mental health policies, services and programmes. It included: National Mental Health Policy (2014), Mental Healthcare Act (2017), NMHP (1982, 1996), District Mental Health Programme (DMHP) and Ayushman Bharat Programme (2018).

### Analysis

The KIIs data were de-identified, transcribed and then translated to English. Thematic Analysis [20] using N-Vivo 12 (©QSR international) was undertaken. The research team comprising of first author and third author, familiarised with the transcripts by going through the data repeatedly. All transcripts were independently reviewed to identify recurrent codes across individuals and groups, by two researchers who have had extensive training in qualitative methodology. After coding the entire data set independently, both researchers reviewed the codes to identify broad thematic areas using an inductive approach. The codes were derived from the data, and findings of the study were then organised under the key themes based on discussions and consensus. The list of codes generated from the data were grouped into potential themes after consensus between the two researchers. Health facility record data was descriptive and close-ended in nature, while policy documents were critically analysed for key features, strengths, and limitations, especially pertaining to addressing stigma and its effect on mental health services delivery.

## Results

### Qualitative key informant interviews

#### Profile of the participants

The age range of sample was 24–56 years. Out of four SUs, three were not aware of their diagnosis while one was reportedly suffering from anxiety and depression. All four of them were taking pharmacological treatment since past one year (from the time of data collection) while only one was seeking therapeutic help.

CHWs had 7–30 years of job experience (Mean = 14.75 years), and the SPs had 2–27 years of job experience (Mean = 11.2 years). Out of the five SPs, one was a psychiatrist [MD (Doctor of Medicine) & MBBS], two were counsellors with post-graduation in psychology [MA (Master of Arts)] while two others were PHC doctors [MBBS (Bachelor of Medicine, Bachelor of Surgery)] with no specialist training in mental health. Six participants worked in primary care facilities, two worked in secondary care facilities and one in a tertiary care facility. Other demographic characteristics are depicted in Table 1.

**Table 1:** Demographics of KII participants (N = 13)

Age (in years), *M* (*SD*)	38.08 (11.61)
Gender (n)	
Female	8
Male	3
Stakeholder	
Type (n)	
Service User (SU)	4
Community Health Worker (CHW)	4
Service Provider (SP)	
Mental Health Specialist	3
Primary Health Care	
Doctors	2
Level of Education	
Secondary Education (15–18 years)	4
Graduate or Post-Graduate	9
Marital status	
Never Married	2
Currently Married	10
Divorced	1
Religious Group	
Hindu	12
Muslim	1
Work status	
Paid work (employee)	11
Paid work (self-employed)	1
Unemployed (health reasons)	1

#### Themes with relevant excerpts

The themes, their descriptions with relevant excerpts from the transcripts of interviews are presented under following headings: (a) Knowledge and perceptions about mental illness; (b) Unequal treatment and feared discrimination; (c) Help seeking behaviours, (d) Health system constraints; and (e) Suggestions and expectations.

#### Knowledge and perceptions about mental illness

All participants (service users and healthcare providers) described similar stigmatizing terms and phrases used by the community in general for person with a mental illness (PWMI), such as Hindi phrases: ‘paagal’ (mad), ‘aalsi’ (lazy), ‘moti-buddhi’ (fat-head), ‘bewkoof’ (idiot), ‘dimag khisak gaya’ (lost mind), and ‘naariyal se sar takraya’‘got hit by a coconut’. English phrases: ‘crazy’, ‘disturbed mind’, and one who doesn’t understand anything, were used as well. Service users and service providers shared that mental illness is not even considered an illness by the community, rather its causes are attributed to supernatural and mystical events.

“And people think that their problems are due to ghosts and paranormal stuff and say no psychiatrist could do anything. They will be cured only by ‘babas’ [holy man] and ‘tantric’ [spiritual practices] by doing some mystical and supernatural activities. People stay away from them and avoid them” (Participant 6, CHW).

“Families do not accept that there is a problem. They say she (patient) is just pretending nothing is happening to her, she will not die…nothing is going to happen to her…she is not willing to do the work. What can be the problem with her mind, she is getting everything, we are fulfilling her basic needs, she is doing this intentionally because she is lazy” (Participant 5, CHW).

Except for one, other service users were not aware about their own diagnosis and described their symptoms in somatic terms of bodily signs such as,

“I used to have body pain and a lot of pain in my head” (Participant 7, SU).

“But my head used to be very hot, from the beginning, my head felt hot…When the head was hot, there used to be lightning like tingling in my brain” (Participant 9, SU).

One service provider narrated an incident where an educated Head of a high school was not able to distinguish between ‘mental illness and mental retardation’.

“There are educated people, like Principal of a school…his reaction was… “Nothing like this is required here, we do not have any need here, we do not give admission to ‘such people’ in our school, there are other schools for ‘such kids’ Madam.”… They do not even know the difference between mental illness and mental retardation” (Participant 4, SP-MHS).

#### Unequal treatment and feared discrimination

SUs described the unequal treatment received from their families and society, on disclosing information about their mental illness. SPs and CHWs shared examples of PWMI not disclosing their condition because of fear of losing their jobs, being perceived as ‘different’ and ‘not able to do anything in their lives’. One SP elucidated several incidents where her colleagues (other health professionals working in the same hospital) were hesitant in visiting the mental health out-patient clinic as patients under fear to be identified and labelled by other colleagues. Four female participants (2 CHWs and 2 SUs) also added that being a female makes one vulnerable and was a risk factor for mental illness as they faced problems due to early marriages, domestic violence, expectations of bearing a male child, pressures of daily chores, and being dictated to by in-laws. One SP added that often women are brought forcefully to the health facility by their in-laws to falsely diagnose them with a mental illness.

“My mother strictly told me not to tell anyone anything about my condition…It got difficult for me to give my college exams after this happened…” (Participant 1, SU).

“My in-laws would say that do this work, that work, you don’t want to and that’s why you act like this. You are not ill…it’s just drama” (Participant 2, SU).

“I haven’t shared anything with them…they won’t understand and get me admitted to an asylum” (Participant 7, SU).

“Real problem is that these people do not have someone with whom they can talk or share their feelings because no one understands them and listens to them” (Participant 11, SP- PHC doctor).

“Doctors around me in the hospital had a lot of issues during covid times and they use to call me over phone, that we need counselling so I use to tell them to come to Out Patient Department (OPD), and they use to say that we can’t come, it’s not such a big issue can you counsel us over phone call?…someone will see us or someone will come to know, I do not want to get the mental health card made, I want to talk to you in general, or I will call you in the evening…” (Participant 4, SP-MHS).

#### Help seeking behaviours

All participants indicated that the first point of contact for them for seeking treatment for mental illnesses were religious and traditional healers, and faith healers. SPs and CHWs mentioned that many patients go to local unregistered practitioners (jhola-chaap/quacks), locally popular religious places, alternative treatment providers who use herbs and ‘special ash’ for treatment, use hot tongs to brand and shock the individuals, or hang some individuals upside down, or make them eat specific non-food items before visiting a mental health outpatient clinic. SUs mentioned that they had visited temples, faith healers, traditional healers, and ‘religious godmen/ holy man’ (*baba*) for years before they visited a doctor or a mental health professional.

“If people have a problem, they do not go to the doctor rather go to pray or worship. They say, “our patient is not crazy, there is a paranormal effect”…then with the blessings of Baba (holy man), by exorcism or by Vibhuti (sacred ash) he will be cured and he has no mental illness. They have the same stigma that neither he has any disease nor any kind of mental illness” (Participant 12, SP-MHS).

“Actually, most of the times patients and their families do not even tell us, one patient recovered after our treatment for depression ended and later he came back again and healed again. One day he said that his mother took him to this baba (holy man) and he burnt him with tongs and hanged him upside down, he had felt better but that never worked fully” (Participant 4, SP-MHS).

“I went to the religious place too, let me tell you, we have a *Baba Bhootnath* temple, it is very famous, we went there and he (the holy man) did his own activities and said that there is nothing more, you do not have to be so nervous, So he had asked me to observe fasts and to distribute Prasad (holy food to others) etc… Didn’t say much and also assured that my mind would be cured. I did not know what was happening to me” (Participant 9, SU).

“Sometimes I use to go to the temple just like that…sometimes, people used to say that if you go there, it will work. I did not believe in something like that, yet I had to consume the special ash due to my family” (Participant 1, SU).

#### Health service and system constraints

SPs described constraints related to human resources, availability of psychotropic medicines, poor awareness about mental health, unavailability of mental health services at primary healthcare level, and lack of mental health training among health professionals. SUs shared issues about unavailability of medicines, distance from the secondary hospital, expenses, and loss of wages during a day-long clinic visit, long waiting hours at the outpatient clinic, and not been able to speak in detail about their problems because their doctors did not have enough time.

“We see around 100 patients in our OPD (Out Patient Department) every day and have not been able to give more than 5–10 minutes per patient…so privacy and time given can’t be ensured. It is very difficult to identify anyone with mental illness in the general OPD in primary health care setup and no time to counsel such patients, even if they are identified…and we don’t have training to identify or tackle mental illness” (Participant 3, SP-PHC doctor).

“There is overcrowding of patients in the hospital, as they have to wait for long time, travel long distances…and lose their daily wages” (Participant 13, SP-MHS).

“I have to buy medicines from private hospitals or pharmacy as most of the time my medicines are not available in government hospital…and its expensive” (Participant 1, SU).

“Medicines for mental illness are not available at district level… because of which there are issues in adherence as poor patients can’t afford it from anywhere else except a government facility. Our pharmacy and hospital staff do not consider psychiatric medicines as important as other medicines, they think counselling is enough and no separate room is required for treatment” (Participant 4, SP-MHS).

#### Suggestions and expectations

Suggestions were made by many participants to address stigma and improve mental health services. These included increasing awareness about mental health within the general public, sharing successful stories of PWMI, increasing training in identification and management of mental disorders for all primary and community health workers, increasing number of trained mental health professionals at primary and secondary level, and organizing door-to-door mental health awareness campaigns.

“Awareness can be spread only by going to the houses of various people and talking to them” (Participant 7, CHW).

“If we organize a camp, share success stories of those who have been cured…they tell themselves that I had such a problem, we went to the doctor, took regular treatment and then healed, then there will be motivation in people… that someone like us could get cured…we are not the only one who has the problem” (Participant 8, CHW).

“Those who have recovered from the illness can set a very good example…in a way to remove stigma, so they can become advocates to talk about it, but situation in hospitals….the challenge will be how to integrate them in the system” (Participant 12, SP-MHS).

“There is a need for making mental health services widely available, free of cost and to remove barriers like stigma. I feel the most important thing to address would be the poor awareness regarding mental health in the community” (Participant 13, SP-MHS).

## Health facility record review

### Primary care setting (Urban Primary Health Centres)

One medical officer was posted and available at each primary healthcare centre. There were no assured mental health related services available (emergency, in-patient or out-patient), no psychotropic medicines, no training facilities for health workers on mental health or any educational materials for the community. No trained mental health professionals or specialists were available for immediate referral. There were no referrals made to secondary hospital in the past one year for treatment of any mental illnesses, no psychotropic medications were prescribed, no counselling provided, no disability benefits related to mental disorders were provided, and there were no support services for caregivers or community-based support for PWMI. There was no designated space for out-patient consultation or counselling services. Only one room which was usually open for all was used for every patient consultation and there was little scope for any privacy. These centres were usually accessible for the local slums, semi-urban communities and villages where they are located. There were no in-patient facilities for patients with a mental illness.

### Secondary care setting (District Hospital)

The Government District Hospital in Faridabad has four mental health professionals employed permanently. Around 70–80 patients are seen in a day in the psychiatry outpatient clinic. Most of the patients consult one psychiatrist at the out-patient clinic. There is no in-patient facility for the psychiatry department. However, in special situations, 2–3 patients with mental disorders can be admitted per month in the internal medicine in-patient facility. One psychiatrist who is the main point of contact in the psychiatry department, provides pharmacological care and approves disability certificates in the out-patient clinic. There is one counsellor who has a post graduate degree in psychology and helps the psychiatrist in the out-patient clinic in preparing the disability certificates, conducts all necessary psychological tests, and provides counselling services. There is a separate room for the counsellor to have sessions in privacy, usually conducted once the outpatient visiting hours are over. There are two psychiatric nurses, one is a psychiatric nurse and the other a community nurse.

Training facilities for professionals in mental health, psychotropic medications, and information and educational materials for the community are available. Electro-convulsive therapies are not available. Specific support services for caregivers, and community are not available.

### Policy document review

#### National Mental Health Programme (1982)

The key objectives of **National Mental Health Programme** (NMHP) were community level services delivery and integration of mental health services with general health care. A district level organisational model (service delivery) was proposed based on a pilot project undertaken in Bellary district in the southern Indian state of Karnataka between 1985 and 1990. The unit of service delivery was PHCs and community health centres (CHCs), however the extent of service delivery was limited [7]. The program had major problems in the form of inadequate budget, and poor clarity about role of the federal government and the state governments. The Bellary model has also been criticised for being largely pharmacologically driven and ignoring psychosocial interventions [7]. This hampered its implementation across the country significantly for many years.

#### District Mental Health Programme (1996)

District Mental Health Programme **(**DMHP) has been implemented in 655 out of a total of 724 districts in India. It is operational in 550 districts [21]. Some of the key components of the programme include training of general physicians for early detection and treatment of mental illness, training and support to general physicians by specialists at the district level, undertaking community level awareness on stigma reduction, and providing additional funding to tertiary care institutions to strengthen infrastructure and train mental health professionals. In 2009, the programme made specific allocations to increase training and availability of mental health workforce. Despite these efforts major gaps remain in availability of mental health services and lack of trained human resources continue to be an area of concern.

With overburdened primary healthcare facilities, medical officers at PHCs showed little interest in undergoing training for mental health care. The training modules were criticised for being lengthy and inappropriate for general physicians [9, 22]. Availability of services at the sub-district level is variable and coverage is not uniform [7]. Many districts do not have the requisite manpower to provide mental health care outside psychiatry departments in district hospitals as per an independent evaluation done by Indian Council of Marketing Research [8].

#### National Mental Health Policy (2014)

The goals of the policy are: (1) to reduce distress, disability, exclusion morbidity and premature mortality associated with mental health problems across lifespan of the person, (2) to enhance understanding of mental health in the country, and (3) to strengthen the leadership in the mental health sector at the national, state, and district levels. The policy has provisions for adequate funding, promotion of mental health and special emphasis on building research capacity. It aims to integrate mental health care into the primary healthcare system and to increase the availability of appropriate housing for homeless and other poorly resourced persons with mental illness living in poverty and deprivation [23].

#### Mental Healthcare Act (2017)

The Mental Healthcare Act (MHA) directs the state to ensure access to affordable and quality mental health services to all persons who need it. MHA 2017 has a broader ambit and adopts a rights-based approach as compared to the older MHA 1987. There is provision for PWMI to provide ‘advance directives’ on how they wish to be cared and be more autonomous. They can also appoint a ‘nominated representative’ who may take care of decisions on their behalf. It had included provisions for mentally ill destitute persons, made electroconvulsive therapy without anaesthesia illegal, prohibited sterilization of PWMI, and decriminalised suicide.

However, criticisms were raised about funding additional bodies to provide oversight of mental health services, like the Central Mental Health Authority and State Mental Health Authority, mentioned in the act, reducing barriers in treatment by revising licensing requirements and difficulties in getting legal remedy given the context of lengthy judicial proceedings in the country [24]. Mental health practitioners have raised concerns about restrictions on providing electroconvulsive therapy, and the difficulty of implementing involuntary institutionalised care for patients who may need it [24–27].

#### Recent developments

In 2018, the government launched a national level programme- *Ayushman Bharat*- to fulfill its commitment for universal health coverage. It has two broad components. First, is a tax funded insurance-based financing scheme for low-income populations that provides cover for a package of secondary and tertiary care hospitalisation procedures and covers about 40% of India’s population. Second, is establishment of Health and Wellness Centres (HWCs). Under the second component, sub-centres and primary healthcare centres will be converted into HWCs that will provide comprehensive primary care services including preventive, promotive, curative, rehabilitative and palliative care. Screening and basic management of mental disorders is included as part of the expanded range of services to be provided at these centres [28]. A new cadre of community level non-physician health workers known as Community Health Officers (CHOs) will play an important role in these centres.

## Discussion

Research shows that contextual factors such as poverty, poor infrastructure, lack of funding, and political instability can all adversely affect equitable mental health service delivery [29]. The situational analysis in the present study aimed to identify stakeholder perspectives and triangulated information gathered from three sources – KIIs of key stakeholders, data (facilities and resources) from some local health facilities, and national and district level programmes for mental health which affect mental health services delivery and is related to stigma alleviation. The results indicate: (a) poor awareness and knowledge about mental illnesses (stigma in both general public and health workers), (b) help-seeking behaviours being driven by local contextual factors (myths, beliefs, and practices), (c) poor availability of mental health services at primary healthcare centres, and inadequate mental health facilities at secondary or tertiary levels, (d) dearth of trained mental health professionals and psychotropic medicines, and (e) inadequate finances, logistics and infrastructure.

### Poor mental health literacy, help seeking behaviours and stigma

Mental health related stigma plays an important role in poor help-seeking and under-reporting of psychological symptoms [1]. Poor knowledge, low mental health literacy levels and issues related with stigma within the community were similar to those observed in previous research in India and other low- and middle-income countries [19, 30, 31]. The data from field notes, KIIs and informal meetings showed that both primary care doctors and ASHAs from both rural and urban settings were not trained in identifying or managing mental illnesses. Although interviews were conducted online with three ASHAs recruited from rural settings because of the pandemic, they had received some amount of training as part of the ongoing SMART programme. However, there was not much difference in training and literacy levels of ASHAs in both urban and rural settings. Although mental health literacy has been included as part of various community based anti-stigma interventions [32–34], stand-alone educational programs are considered ineffective and momentary [35, 36]. One alternative that has yielded promising results is incorporating service user presentations and photographic recovery narratives into structure mental healthcare training for primary care workers: in Nepal this strategy showed a reduction in primary care workers’ stigma and an improvement in accurate detection and diagnosis of mental illness in primary care settings [37].

Knowledge about mental illnesses in India is influenced by several cultural factors resulting in a majority of the community people looking for a cure in traditional, religious or faith related healing practices [10]. The general public do not categorise mental illness as an illness rather hold false beliefs, and attribute it to bad sins and supernatural reasons [2, 4, 31], which were evident in the KIIs. Barriers in help-seeking are not only limited to stigma and cultural beliefs. Research suggests additional obstacles such as travel distance for service utilization, poverty, and lack of public transport lead to poor help seeking behaviours [31]. Lack of awareness, misconceptions about common signs and symptoms of mental disorders and scarcity of psychotropic medications are also reasons for poor-help seeking.

Discrimination and stigmatizing behaviours against PWMI further impact the use of health care system by PWMI. Thus, understanding local perspectives (cultural practices, beliefs and behaviours) is important for a culturally sensitive healthcare system for the community [38, 39]. Delayed health-seeking behaviour, illiteracy, cultural and geographic distribution of people are causes for psychiatric morbidity [40, 41]. Medical professionals share a high proportion of misconceptions and have discriminatory attitude toward psychiatry and PWMI [40, 42]. Levels of mental health literacy is not only poor amongst the community members, but even amongst the health care professionals working in tertiary care hospitals [43], which was also described from a secondary care setting in Faridabad [44].

Poor mental health literacy and discriminatory behaviours against PWMI contribute to stigma. Stigma can be understood as a problem of knowledge, attitudes and behaviour [45]. PWMI suffer from twofold problems, dealing with their symptoms and misunderstandings of society about mental disorders resulting in stigma [46]. A multisite study conducted in Southern India (collaborative community care for people with schizophrenia in India (COPSI Trial) observed that internalized stigma was more of a challenge as compared to societal stigma [47]. The feared discrimination seen in the present study is similar to ‘others finding out’ (key theme identified in COPSI Trial) despite the differences in study site and setting.

### Inadequate mental health services and facilities

The availability of mental health resources in India is poor as enumerated in the Project Atlas of World Health Organization [48]. Haldar and colleagues [44] reported on the psychiatric burden in a sub-district secondary level hospital in Faridabad district. The researchers reported that few females sought mental health care. The link between the primary and secondary or tertiary care facilities was also poor. Our health facility review showed that not even a single referral was made from the primary healthcare centres to the district hospital in the past one-year. The reasons cited for this were lack of training in identifying and managing PWMI at PHC settings, inadequate time for the PHC doctors to listen to the psychological complaints of PWMI, lack of privacy and time to assess patients for mental health conditions, stigma and feared discrimination in the community about mental illness and PWMI that prevents them from help-seeking.

Despite evidence that task shifting of mental health care delivery is feasible and primary healthcare doctors could effectively diagnose and treat MI [30, 49], DMHP has not considered setting up psychiatric units at primary care level and training the primary and community health workers in mental health. Decentralization and integrated primary mental healthcare with a task-sharing approach could address both scarcity of trained mental health professionals and lessen treatment gap for MI [50]. The services provided in the District hospital in our study were also not optimum for a specialist centre. Excessive number of patients, few mental health professionals, lack of administrative support for improving mental health infrastructure in the hospital are some of the factors adversely affecting mental health services delivery at the district hospital. Mental health professionals had little time to discuss about diagnosis, prognosis and stigma with their patients. During our field visits, we observed many patients were satisfied with getting five minutes of psychiatrist’s attention after waiting for a long time and did not bother to ask questions about their treatment or diagnosis (example, 3 SUs in our study sample did not know about their diagnosis). The specialist room was quite small, with no privacy and a fresh intake was given maximum of 5–10 min. More focus was seen on prescribing psychotropic medications, out of which many were not available even at the district level pharmacy (as mentioned by SUs and SPs).

### Mental health policies and global mental health

Though the NMHP and the Mental Health Policy propose a decentralized model of care, there are vast differences in implementation of those across the country. The condition of mental health care services in Faridabad need improvement. Setting up of mental health units only at district hospital may not be sufficient, as envisioned in DMHP [44]. There needs to be community participation, involvement of civil society organisations, and regular training of community and primary healthcare workers in mental health, for operationalizing the objectives [7]. Community-based anti-stigma campaigns could also involve traditional and faith healers who are often the first point of contact for PWMI.

The Lancet Commission on Global Mental Health and Sustainable Development [51] stated that the quality and funding for mental health services was worse than for physical health services. The redistribution of mental health budgets from large hospitals to primary and community-based facilities was also suggested. Equal rights to health, inclusion, education [52], employment and social protection and participation in political and public life for PWMI has been mentioned in World Health Organization Comprehensive Mental Health Action Plan [53]. All the policy documents have highlighted the need to alleviate stigma related to mental health and designing community-appropriate stigma reduction programs (Table 2). Policy documents also mention ensuring funding required to improve mental health awareness and literacy. Recent developments have mentioned the need to involve frontline health workers in the effective implementation of anti-stigma techniques. However, development and implementation of stigma reduction programs are neglected and underappreciated despite stigma having been recognized as a key focus in the policy documents.

Use of only educational materials such as pamphlets or brochures are outdated unless complemented by other strategies. Community engagement, participation and involvement of PWMI have been found to be effective in mental health stigma reduction and uptake of services [32, 54, 55]. It is important to explore the experiences of PWMI and their families from human centered and empathetic viewpoint as defined by the human centered design approach to reduce stigma [56]. The latest Lancet Commission on ending stigma and discrimination in mental health recommends using social contact approach and involving the PWMI as the key change agents for reducing stigma [6].

**Table 2:** Policy documents highlighting stigma related to mental health

Policy Document	Provisions related to stigma reduction	Actual Text (excerpts)
National Mental Health Policy, 2014	Mentions stigma as a cross-cutting issues that can impact attaining policy goals Specifically mentions need to address stigma in suicide prevention	*“Persons with mental health problems face stigma and discrimination in many ways…. Government, opinion-makers, media, and community leaders should encourage discussions for better understanding of the nature of mental health problems. There is need for compassion and responsibility in our interaction with persons affected with mental health problems instead of stigmatising such persons” p6*
Mental Healthcare Act, 2017	Mental health awareness and stigma reduction mentioned as a duty of the appropriate government Mandates governments to design appropriate programmes for stigma reduction and ensure appropriate funding.	*“Creating awareness about mental health and illness and reducing stigma associated with mental illness.” (Chapter VI-Duties of Appropriate Government, p2)* *“The programmes to reduce stigma associated with mental illness are planned, designed, funded, and implemented in an effective manner.” (Chapter VI-Duties of Appropriate Government, p17)*
National Mental Health Programme (1982)	The District Mental Health programme includes stigma reduction as a key component in its IEC activities after 1996.	*“The DMHP envisages a community-based approach to the problem, which includes …… Increase awareness & reduce stigma related to Mental Health problems.” p2*
Recent Developments- Operational Guidelines for Mental, Neurological and Substance Use Disorders (MNS) care at Health and Wellness Centres (HWCs), Ayushman Bharat (2018)	Community level awareness activities for stigma reduction to be undertaken by frontline worker team at Health and Wellness Centres (HWCs).	*“Community level Health Promotion interventions and improving mental health literacy that enables an understanding of mental health, common symptoms, risk factors/causes of disorders, treatment, reduction of stigma and discrimination, and of techniques such as psychological first aid, and self-care.” p6* *“Awareness building and stigma and discrimination reduction activities through IEC and community mobilisation will be conducted by ASHA/ MPW in the community.” p7*

### Recommendations

Task shifting is practical, feasible and beneficial in context of scarcity of trained mental health professionals and mental health facilities not being available at primary care level [30]. Community health workers can play a vital role in identifying PWMI and promoting positive attitudes amongst the community [4]. Maulik et al. [30] implemented task sharing by training primary care doctors and community health workers in screening, identifying and caring for PWMI using technology-enabled mental health service delivery model and anti-stigma campaign for rural India. This large complex intervention study from an LMIC (SMART Mental Health) could lead the way for equitable and accessible mental health services, which is the key objective under Mental Health Action Plan 2013–2030. SMART Mental Health intervention has been scaled up and currently ongoing in each state of northern and southern India respectively, as a cluster randomised controlled trial [12] where (a) use of mobile technology based electronic decision support system is being used to facilitate mental health care delivery, (b) task sharing is used by training community health workers (ASHAs) in screening of common mental health disorders, making referrals to PHC doctors, and (c) a parallel anti-stigma campaign is being conducted which involves a mix of print materials, animation videos, live and recorded drama, social contact and awareness videos. Active involvement of health and social care staff, employers, policy makers, all media organizations and PWMI of mental health conditions is required in reduction of stigma [6].

Though research about stigma has been absent from large parts of Northern and Eastern India [57], the current study provides information on the scenario of stigma, service utilization and provisions related to mental health in one district in Northern India. This can have implications in future research, developing and implementing of mental health related stigma reduction interventions at various levels with primary and community health workers, general community, and mental health professionals.

### Limitations

A key limitation of our study is that policy makers and caregivers of PWMI could not be contacted as key stakeholders due to the COVID outbreak. The current study made use of online consenting and phone call interviews for data collection during the COVID-19 pandemic. Such modalities could be explored or evaluated further. However, phone call interviews have their own challenges. The pandemic had impacted our purposive sample as we couldn’t interview ASHAs working at urban settings. However, our field observations had indicated that both rural and urban ASHAs’ level of mental health literacy and training are poor. The findings from facility-based review are also difficult to generalize as the availability of mental health services may vary across different regions. In this study, we looked at only key mental health policy and programme related documents. However, a more critical policy review could involve exploring other related papers and research from individual studies, which was beyond the scope of this site-specific situational analysis.

## Conclusion

This study highlights the importance of conducting situational analysis to elucidate contextual factors related to mental health related stigma, service utilization and delivery. We reported poor awareness and knowledge about mental illnesses, belief in faith and traditional healers, scarcity of resources (medicines, trained professionals and mental health inpatient and outpatient clinics), poor access to appropriate mental health facilities, and high costs for seeking mental health care. There is a critical gap between mental health related provisions in policy documents and its implementation at primary and district level.

## Data Availability

The datasets used and/or analyzed during the current study are available from the corresponding author on reasonable request.
